# The Role of Nuclear Insulin and IGF1 Receptors in Metabolism and Cancer

**DOI:** 10.3390/biom11040531

**Published:** 2021-04-02

**Authors:** Haim Werner, Rive Sarfstein, Zvi Laron

**Affiliations:** 1 Department of Human Molecular Genetics and Biochemistry, Sackler School of Medicine, Tel Aviv University, Tel Aviv 69978, Israel; rives@tauex.tau.ac.il; 2 Shalom and Varda Yoran Institute for Human Genome Research, Tel Aviv University, Tel Aviv 69978, Israel; 3 Endocrine and Diabetes Research Unit, Schneider Children’s Medical Center, Petah Tikva 49292, Israel; laronz@clalit.org.il

**Keywords:** insulin-like growth factor-1 (IGF1), IGF1 receptor, insulin receptor, transcription factors, cancer, cell nucleus

## Abstract

Insulin (InsR) and insulin-like growth factor-1 (IGF1R) receptors mediate the metabolic and growth-promoting actions of insulin and IGF1/IGF2, respectively. Evidence accumulated in recent years indicates that, in addition to their typical cell-surface localization pattern and ligand-activated mechanism of action, InsR and IGF1R are present in the cell nucleus of both normal and transformed cells. Nuclear translocation seems to involve interaction with a small, ubiquitin-like modifier protein (SUMO-1), although this modification is not always a prerequisite. Nuclear InsR and IGF1R exhibit a number of biological activities that classically fit within the definition of transcription factors. These nuclear activities include, among others, sequence-specific DNA binding and transcriptional control. Of particular interest, nuclear IGF1R was capable of binding and stimulating its cognate gene promoter. The physiological relevance of this autoregulatory mechanism needs to be further investigated. In addition to its nuclear localization, studies have identified IGF1R in the Golgi apparatus, and this particular distribution correlated with a migratory phenotype. In summary, the newly described roles of InsR and IGF1R as gene regulators, in concert with their atypical pattern of subcellular distribution, add a further layer of complexity to traditional models of cell signaling. Furthermore, and in view of the emerging role of IGF1R as a potential therapeutic target, a better understanding of the mechanisms responsible for nuclear IGF1R transport and identification of IGF1R interactors might help optimize target directed therapies in oncology.

## 1. Overview of the Insulin-Like Growth Factor System

The insulin-like growth factors (IGFs) are a family of mitogenic growth factors, cell-surface receptors and binding proteins that are involved in normal growth, development and differentiation of most organs and tissues as well as in a number of pathological conditions [1,2]. IGFs developed early in evolution, probably as regulators of cellular proliferation in relation to nutrient availability [3,4]. The role of IGF1 as the mediator of the growth hormone (GH)-stimulated incorporation of sulfate into cartilage was demonstrated more than 60 years ago [5]. The specific, GH-activated serum factor that was originally termed ‘sulfation factor’ and then ‘somatomedin-C’ is now accepted as IGF1 [6]. The IGF system comprises two ligands, IGF1 and IGF2. At the cellular level, IGF1 is regarded as a key progression factor required to traverse the cell cycle [7]. Circulating IGF1 levels are primarily dependent on liver production, which is controlled by pituitary-derived GH. In addition to its classical endocrine role, many extrahepatic tissues (e.g., brain, kidney, stomach, etc) display the biosynthetic machinery necessary to produce IGF1. Locally-produced IGF1 exhibits paracrine and autocrine modes of action [8].

IGF2 shares a ~62% amino acid homology with IGF1. Ontogenetic studies in rodents have demonstrated that IGF2 is expressed at very high levels during the prenatal period [1,4]. On the other hand, postnatal stages are characterized by a drastic reduction in circulating and tissue IGF2 levels. These early observations led to an erroneous generalization of the roles of IGF2 and IGF1 as fetal and pubertal growth factors, respectively. In humans, however, both ligands are produced from prenatal to postnatal stages and, in fact, endocrine IGF2 dosages are higher than IGF1 in adults [4]. The physiological role of IGF2, as well as its dependence on GH production, are still controversial topics.

Both IGF1 and IGF2 activate a common receptor, the IGF1 receptor (IGF1R), which signals mitogenic, antiapoptotic and pro-survival activities [9]. The IGF1R is a heterotetrameric, cell-surface tyrosine kinase receptor that is structurally and evolutionarily related to the insulin receptor (InsR) [10,11]. The IGF1R is coupled to several intracellular second messenger pathways, including the ras-raf-MAPK and PI3K-AKT signaling cascades [12]. The IGF1R is vital for cell survival, as illustrated by the lethal phenotype of mice in which the *IGF1R* gene was disrupted by homologous recombination [13].

The IGF system includes, in addition, a series of six high-affinity IGF binding proteins (IGFBP1-6) that carry IGF1 and IGF2, but not insulin, in the circulation and extracellular spaces. For the most part, the IGFBPs inhibit IGF’s metabolic and proliferative actions, although some IGFBPs display IGF-potentiating effects [14,15]. In serum, the vast majority of circulating IGF1 and IGF2 is found in a ternary complex with IGFBP3 and an acid labile subunit [16]. This complex modulates IGF action by protecting the growth factors from proteolysis and prolonging their half lives in body fluids [17]. Finally, some IGFBPs exert a number of biological effects in an IGF-independent manner [18].

## 2. Classical Concepts on Hormonal Signaling and Internalization

The classical view on hormonal signaling endorses the dogma that this intercellular communication system constitutes an unidirectional cascade that initiates with the binding of an extracellular ligand to a specific, or cognate, receptor or to closely related receptors, followed by receptor activation, usually in the form of a phosphorylation event. This energetic state is then transmitted downstream to a series of cytoplasmic proteins that, in a sequential and highly orderly manner, convey this signal to the nucleus, where genes are turned ‘on’ or ‘off’. In turn, this cascade leads to different, often opposite, biological activities. There is a certain degree of crosstalk between ligands and receptors, but the hormonal signaling is primarily unidirectional, i.e., from cell surface to nucleus.

Receptor internalization, or endocytosis, has been commonly regarded as a mechanism required for receptor degradation, usually in endosomes and lysosomes. Most of the trafficking pathways and players involved in this process have been extensively characterized over the years, including caveolins, clathrin-coated pits, etc. [19,20]. It is nowadays clear that receptor endocytosis is, in fact, also critical for the formation of signaling complexes, termed signalosomes, and for fine tuning of hormonal signals [21]. Specifically, internalized receptors are capable of engaging different lysosomal and recycling paths, with ensuing attenuation or, alternatively, enhancement of the hormonal signal [22]. Hence, internalization is indispensably required for proper receptor homeostasis and regulation.

## 3. Historical Aspects of Nuclear InsR/IGF1R Translocation

Early experimental evidence of nuclear InsR presence was provided forty-five years ago by Goldfine and Smith [23]. Using purified nuclei from rat livers, they demonstrated a rapid and reversible binding of labeled insulin to this fraction. These initial analyses were subsequently expanded by Goldfine and colleagues, who were able to identify the nuclear envelope as the major site of insulin binding in nuclei [24,25]. Furthermore, authors established that intracellular binding sites were immunologically distinct from those on the plasma membrane. Of historical interest, Goldfine stated in his paper that “the exact physiological significance of the cellular uptake and nuclear binding of insulin is not known”. Following these pioneering studies, two independent reports by Horvath [26] and Bergeron et al. [27] corroborated the specific binding of insulin to liver-derived nuclei and Golgi fractions.

In terms of attempts to elucidate the mechanistic aspects associated with nuclear InsR presence, electron microscopy allowed Podlecki et al. [28] in 1987 to visualize InsR internalization. Immunofluorescence analyses conducted by Chen et al. [29] revealed that IGF1R was present in the nucleus of renal epithelial cells and that estrogen treatment led to a doubling of nuclear IGF1 binding. In addition to insulin and IGF1 receptors, early studies also explored the nuclear presence of cytoplasmic InsR/IGF1R target molecules. The laboratory of Renato Baserga reported that IRS-1, -2 and -3 translocate to the nucleus and nucleoli in several types of cells following induction by an activated IGF1R or certain oncogenes [30]. Of importance, an intact IGF1R tyrosine kinase domain was a key prerequisite for nuclear translocation of IRS-1 and IRS-2. Mutations in this intracellular IGF1R domain abrogated IRS transport. Finally, additional cytoplasmatic mediators involved in InsR/IGF1R signaling, including PI3K, AKT and MAPKs, were also identified in nuclei [31]. An important ‘take-home message’ from these pioneering studies would be that early distinctions between membranal, cytoplasmic and nuclear components must be re-evaluated in light of available modern technologies. Current views support a more dynamic model of regulated translocation between compartments. The studies described in this article highlight novel signaling pathways and additional levels of biological regulation.

## 4. Proteomic Profiling Identified the IGF1R in Nuclear Fractions of Breast Cancer Cells

Proteomic profiling analyses aimed at characterizing the entire collection of *IGF1R* promoter-binding proteins led us to the identification of the IGF1R in cell nucleus. Briefly, human *IGF1R* promoter fragments were labeled with biotin, followed by incubation with nuclear extracts of breast cancer cells, and purified over streptavidin beads. Bioinformatic analyses of proteins in eluates allowed us to identify approximately 100 nuclear proteins that were either directly linked to the *IGF1R* gene promoter or, alternatively, part of multimeric protein complexes associated with this DNA region [32].

Most of the identified *IGF1R* promoter-binding proteins were typical nuclear proteins, including DNA helicases, DNA repair proteins, elongation factors, RNA splicing proteins, etc. The presence of IGF1R in association with its cognate promoter was unexpected as it contradicted established dogmatic notions [33]. While IGF1R was detected in nuclei and perinuclear areas of both estrogen receptor-α (ER α)-expressing and ER α-depleted cells, differences were seen in the ability of nuclear IGF1R to bind its own promoter. Thus, IGF1R was detected in association with the *IGF1R* promoter region in ER α-depleted, but not ER α-containing, breast cancer cells. These results are consistent with competition between IGF1R and ER α proteins for binding to consensus GC elements in the *IGF1R* promoter [12,34]. In other words, IGF1R was able to bind its cognate gene promoter in cells with reduced ERα levels and, as a consequence, reduced ERα binding to the *IGF1R* gene. The autoregulation of *IGF1R* expression by nuclear IGF1R is described below.

## 5. Potential Mechanisms of InsR/IGF1R Nuclear Translocation

Nuclear translocation of InsR and IGF1R is attained via proteosomal, lysosomal and endocytic pathways [35]. Of note, these paths are also operative in the process of InsR/IGF1R degradation. Thus, dansylcadaverine, a clathrin-dependent endocytosis inhibitor, prevented nuclear translocation of IGF1R [36]. Inhibition of nuclear import had a marked impact on cell proliferation and migration, as revealed by XTT and scratch assays, respectively. Furthermore, combined treatment of dansylcadaverine and a specific small MW IGF1R inhibitor had a synergistic effect on proliferation. Given the emerging role of IGF1R as a therapeutic target in oncology, these results may be of translational relevance.

**Figure 1:**
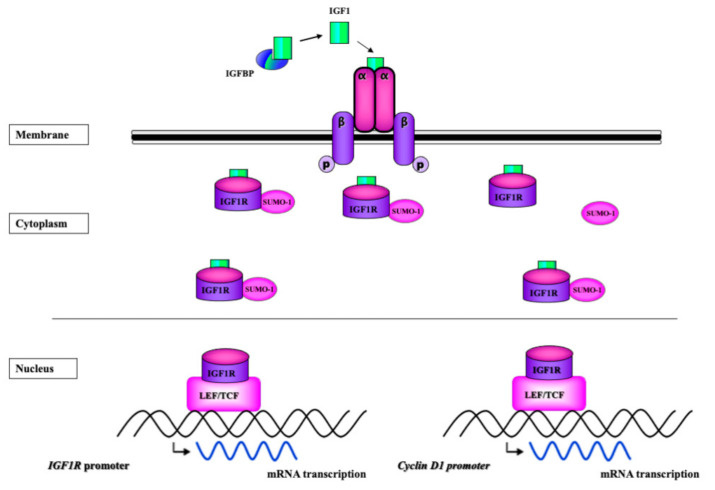
Schematic representation of IGF1R nuclear translocation. Nuclear IGF1R internalization is usually, but not necessarily, regarded as a ligand-dependent, SUMO-1-mediated translocation event. The intracellular domain of IGF1R includes three lysine residues that appear to be involved in the interaction with SUMO-1. Nuclear IGF1R is capable of binding DNA enhancer regions in a sequence-specific manner and as part of multimeric protein complexes. SUMOylated IGF1R binds to a LEF/TCF binding site in the *cyclin D1* promoter, leading to enhanced gene activation. Nuclear IGF1R was shown to autoregulate expression of its own gene via interaction with LEF-TCF.

Evidence in support of the hypothesis that IGF1R is transported to the nucleus along microtubules was provided by studies showing that IGF1R co-localizes with α-tubulin [37]. Importin-β, a key player in nuclear mobilization, was demonstrated to co-immunoprecipitate with IGF1R. In this context, RanBP2, a SUMO E3 ligase located at the nuclear pore complex, was shown to bind IGF1R [38]. Moreover, RanBP2 expression enhanced IGF1R SUMOylation and nuclear IGF1R levels. Three functional SUMOylation sites were identified in the human IGF1R sequence [39]. Lysines 1055 and 1130 are conserved in the IGF1R and InsR, whereas lysine 1150 is also conserved in the epidermal growth factor receptor (EGFR). A schematic representation of the SUMO-dependent nuclear IGF1R translocation is presented in Figure 1.

The question of whether InsR/IGF1R SUMOylation constitutes a mandatory prerequisite for nuclear internalization is still a matter of controversy [40]. Sehat et al. reported that SUMOylation is essential for IGF1R nuclear transport [39]. In contrast, Aleksic et al. generated data showing that SUMOylation is not a vital requisite [36]. Interestingly, evidence has been provided showing that SUMOylation is necessary for IGF1R-stimulated proliferation but is not critical for receptor internalization [41]. The use of a cellular model that prevents IGF1R SUMOylation allowed Deng et al. [42] to demonstrate that accumulated nuclear IGF1R leads to high expression of SUMO-conjugating enzyme Ubc9. The issue of whether receptor internalization is an IGF ligand-dependent process has not yet been solved. While Aleksic et al. [36] showed that IGF1-stimulated IGF1R activation is indeed required for nuclear translocation, our data suggested that IGF1R internalization may occur in a ligand-independent manner [33,43].

## 6. Transcriptional Activity of InsR and IGF1R

The ability of IGF1R to directly bind DNA was demonstrated by means of electrophoretic mobility shift assays using biotin-labeled oligonucleotides [39]. At a genomic level, chromatin immunoprecipitation (ChIP) experiments revealed that more than ~80% of IGF1R-enriched regions were intergenic (i.e., distal from any annotated gene). In addition, approximately 6% of IGF1R-enriched regions were located in introns and 6% in exons. Therefore, the data are in agreement with the concept that nuclear IGF1R is capable of specifically binding DNA enhancer regions. The ability of InsR and IGF1R to function as transcriptional activators has been confirmed in recent years by a number of laboratories. The role of InsR/IGF1R as gene regulators adds a further layer of complexity to classical models of cell signaling by: (1) identifying tyrosine kinase cell-surface receptors in atypical cellular compartments and (2) discovering new activities formerly associated solely with transcription factors [40].

In order to examine IGF1R subcellular distribution and to gain further insight into its transcriptional role, Jamwal et al. [44] recently generated a library of IGF1R deletion and point mutants and provided evidence that nuclear IGF1R presence is essentially defined by its cytoplasmic portion. Authors identified a cross-talk between IGF1R and the Wnt/β-catenin pathways and showed that IGF1R upregulated TCF-mediated β-catenin transcriptional activity. Likewise, nuclear IGF1R was associated with LEF-1, a transcription factor involved in Wnt signaling [37]. Consistent with the interplay between IGF1R and Wnt/β-catenin, we have also identified LEF-1 in association with the *IGF1R* promoter [32]. Of interest, nuclear IGF1R was shown to phosphorylate histone H3 at Tyr 41 in HeLa cells, an event that stabilized the binding of Brg1 chromatin remodeling protein to histone H3. Moreover, the SNAI2 oncogene was identified as a target gene for nuclear IGF1R action, with potentially important consequences in cancer invasion and metastasis [45]. In summary, the reported nuclear actions of IGF1R fall within the spectrum of activities usually associated with transcription factors.

## 7. Autoregulation of *IGF1R* Gene Expression by IGF1R

**Figure 2:**
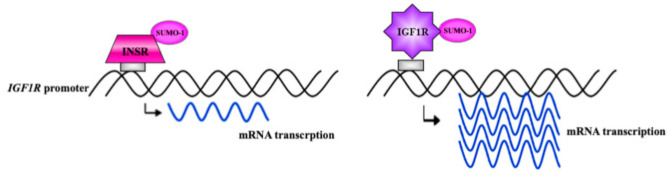
Comparison between the roles of InsR and IGF1R in transcriptional regulation of the *IGF1R* gene. InsR and IGF1R were shown to translocate to the cell nucleus and bind to the *IGF1R* gene promoter. However, whereas IGF1R stimulated its cognate promoter, InsR inhibited *IGF1R* promoter activity. The impact of the differential regulation of *IGF1R* gene expression by InsR and IGF1R is yet to be elucidated.

The identification of IGF1R in association with its cognate promoter raised the question of whether *IGF1R* transcription might be regulated by nuclear receptor concentrations. Co-transfection experiments in breast cancer cell lines using an IGF1R expression vector along with an *IGF1R* promoter-luciferase reporter construct demonstrated that IGF1R stimulated the activity of its cognate promoter. In correlation with the extent of IGF1R binding to promoter DNA, the level of promoter activity stimulation was higher in ER α-depleted than in ER α-containing cells [33]. Of interest, nuclear IGF1R and InsR display diametrically opposite activities in the context of *IGF1R* promoter regulation. Thus, whereas IGF1R stimulated its cognate promoter, InsR inhibited *IGF1R* promoter activity (Figure 2).

Characterization of the human *IGF1R* promoter allowed for the identification of cis-elements and trans-acting factors that govern *IGF1R* gene expression in normal and disease conditions [12]. Our analyses provided evidence that *IGF1R* gene transcription is achieved via physical and functional interactions between positively- and negatively-acting transcription factors [9]. The finding that IGF1R was capable of stimulating the *IGF1R* promoter, while InsR inhibited it, is consistent with the fact that insulin has an inhibitory effect on the *IGF1R* gene. We postulated that negative regulation of *IGF1R* biosynthesis, with ensuing reduced activation of the IGF1R signaling pathway, might favor insulin-mediated differentiative activities. Finally, the physio-pathological implications of the interplay between InsR and IGF1R in regulation of *IGF1R* gene expression are yet to be elucidated. It is unknown whether *IGF1R* autoregulation constitutes a general mechanism of gene regulation. Recent studies, however, have identified a similar paradigm of gene regulation in the context of the *INSR* gene.

## 8. Autoregulation of *InsR* Gene Expression by InsR

Co-transfection experiments in breast cancer cell lines harboring specific disruptions of either the IGF1R or InsR allowed us to investigate the impact of nuclear InsR on *InsR* gene expression [46]. Studies provided evidence that InsR-A strongly stimulated the *InsR* promoter. In addition, data identified complex functional and physical interactions between tumor suppressor p53 and the InsR pathway. The translational implications of this interplay, particularly in regard to InsR/IGF1R targeted therapies, needs to be critically assessed in a clinical setting.

## 9. Is Nuclear InsR/IGF1R Translocation Limited to Malignant Cells?

Most experimental evidence gathered in recent years on the role of nuclear InsR/IGF1R was generated using cancer cell lines as well as freshly obtained tumors or archival specimens. Despite the fact that most early evidence on InsR/IGF1R internalization emanated from non-transformed cells, the question of whether nuclear InsR/IGF1R translocation constitutes a common physiological process in normal cells has not yet been explored in a systematic fashion. As alluded to above, initial proof of insulin binding to nuclear fractions was demonstrated in rat livers [23,24,25,26,27], while nuclear IGF1R was visualized in renal epithelial cells [29]. It is tempting to speculate that in spite of solid evidence of nuclear InsR/IGF1R presence in normal cells, the existence of cell-surface tyrosine-kinase receptors in the nucleus was regarded as an ‘anti-dogmatic’ view for many years.

We have recently addressed the hypothesis that nuclear transport is not restricted to malignant cells and, in fact, may constitute a novel physiologically-relevant cellular mechanism. Using cell fractionation and confocal microscopy we demonstrated that nuclear IGF1R translocation also occurs in normal, non-transformed cells, including primary diploid fibroblasts and immortalized non-malignant mammary gland-derived cells (e.g., MCF10A) [43]. Of interest, we noticed the accumulation of IGF1R in perinuclear areas of benign MCF10A cells. In addition, small interfering RNA (siRNA) against IGF1R led to a decrease in IGF1R levels in the cytoplasm but not in the nucleus. We assume that this differential pattern was due to the mechanism of action of siRNA that only involves mRNA degradation in the cytoplasm. Thus, the nuclear presence of cell-surface receptors seems to provide an additional layer of biological regulation under normal conditions.

Recent mass spectrometry (MS)-based proteomic analyses, designed to identify nuclear IGF1R interactors, allowed us to detect a series of novel proteins that have not been previously linked to the IGF1 signaling pathway [47] (Table 1). Briefly, experiments consisted of immunoprecipitation of the nuclear fractions of benign MCF10A breast cells using an IGF1R antibody, followed by MS analysis of candidate interactors. We were able to detect 18 proteins that interacted, either directly or as part of multimeric complexes, with nuclear IGF1R. Some of the identified proteins (e.g., TJP2, VAPB, TJP1, TUBB, etc) appear to be involved in structural aspects of nuclear function, including tight junction formation.

## 10. Nuclear Localization of Hybrid InsR/IGF1R

InsR and IGF1R can dimerize to form hybrid InsR/IGF1R, composed of one α−β InsR hemireceptor linked to an α−β IGF1R hemireceptor [48,49,50]. The presence of hybrid receptors seems to correlate with high expression of the individual partners and is also associated with crosstalk of insulin and IGF1 signaling [51]. Reciprocal immunoprecipitation of the nuclear fractions of corneal epithelial cell lysates identified hybrid receptors in this subcellular portion [52]. Nuclear hybrid receptors were mainly localized in the chromatin-bound fraction, suggesting a role in gene regulation. In addition, genome-wide ChIP assays confirmed the interaction of IGF1R and InsR with DNA. Finally, Gene Ontology analyses revealed that the vast majority of hybrid InsR/IGF1R target genes were involved in cell-cell adhesion processes.

The role of insulin in mediating the nuclear accumulation of hybrid InsR/IGF1R was recently investigated in corneal epithelial cells [53]. Stress-induced nuclear import of hybrid receptors was correlated with partial cell cycle arrest and abrogation of mitochondrial respiration. Insulin, but not IGF1, restored cell cycle dynamics and proper metabolic function. Therefore, the data support an important role for insulin in controlling corneal epithelial cells’ homeostasis, with potential relevance in diabetes and wound healing.

## 11. IGF1R Localization in the Golgi Apparatus

A recent study from the laboratory of Rosemary O’Connor employed a novel approach to address aspects related to IGF1R activity in malignant cells [54]. Using mass spectroscopy and phospho-specific antibodies, the authors demonstrated that IGF1R was autophosphorylated on residues Tyr^1250/1251^ under basal conditions. The impact of this phosphorylation event was investigated using a phospho-mimetic mutant form of IGF1R. This mutant was shown to be more rapidly internalized than the wild-type IGF1R. Upon IGF1 stimulation, both the wild-type and phospho-mimetic version of IGF1R rapidly accumulated within the Golgi apparatus. On the other hand, a phospho-impaired version of the receptor was retained in the plasma membrane. The localization of IGF1R in the Golgi correlated with a migratory phenotype. Taken together, the data are consistent with the idea that Tyr^1250/1251^ phosphorylation drives the translocation of IGF1R to the Golgi apparatus, where it might contribute to invasiveness. Of clinical relevance, these results may partially explain the lack of efficacy of current approaches aimed at targeting the cell-surface IGF1R for therapeutic purposes.

Using a combination of proteomic analyses along with silencing experiments, we discovered a complex, bi-directional interplay between nuclear IGF1R and nucleolar protein NOM1 [47]. The nucleolar NOM1 molecule belongs to a family of proteins that contain the middle domain of eukaryotic initiation factor 4G (MIF4G) and/or protein-protein interaction module (MA3), and function in translation, proliferation and transformation [55,56]. NOM1 localizes predominantly to the nucleolus and interacts with protein phosphatase-1, an essential eukaryotic serine/threonine phosphatase required for a number of cellular processes, including mitogenesis [57]. In summary, identification of nuclear IGF1R interactors may help generate new information on the role of IGF1R in the nucleus and additional subcellular compartments.

## 12. Nuclear Localization Is a Common Theme among Tyrosine Kinase Receptors

Endocytosis and endosomal sorting are involved in the nuclear import of additional tyrosine kinase receptors [58]. EGFR was detected in the nucleus of several tissues and cell lines, and its nuclear presence correlated with strong proliferative activity [59]. Nuclear EGFR is associated with the *cyclin D1* gene promoter, suggesting that it may serve as a transcriptional regulator. Furthermore, a number of studies reported that full-length EGFR is involved in a variety of gene regulatory activities, including transcription, DNA replication and DNA repair [60,61].

Likewise, the platelet-derived growth factor receptor (PDGFRα) was shown to be present in nuclear fractions of alveolar rhabdomyosarcoma along with IGF1R [62]. Authors reported a dynamic pattern of expression between cell surface and intracellular compartments. An additional example of tyrosine kinase nuclear import was provided by the fibroblast growth factor receptor (FGFR1), which forms a complex with orphan nuclear receptor Nurr1 and binds to the tyrosine hydroxylase (TH) promoter (the rate limiting enzyme in dopamine synthesis) [63]. This nuclear partnership constitutes a new mechanism for *TH* gene regulation, with relevance in dopaminergic neuron development. It is reasonable to conclude that nuclear import constitutes a widespread mechanism among cell surface tyrosine kinase receptors. This machinery impinges upon multiple physiological and pathological processes and largely expands the spectrum of classical intracellular communication paths.

## 13. The Role of Nuclear InsR/IGF1R in Cancer

The effect of nuclear InsR/IGF1R on the mitogenic capacity of cells and, more specifically, the impact of this pattern of subcellular distribution on our ability to target the IGF1R for therapeutic purposes, has been the topic of major interest in recent years [64]. The relevance of nuclear IGF1R in terms of tumor aggressiveness can be inferred from the fact that the inhibition of nuclear IGF1R import correlated with reduced proliferative potential [36]. Aleksic et al. reported that nuclear IGF1R was present in renal cancer cells, preinvasive breast lesions and non-malignant tissues associated with a high proliferative index. Furthermore, nuclear staining was correlated with an adverse prognosis in renal cancer. Similarly, nuclear IGF1R localization in alveolar rhabdomyosarcoma was associated with an aggressive phenotype [62]. Consistently, IGF1R silencing reduced the ability of tumor cells to form colonies.

The association between nuclear IGF1R and clinical outcome in pediatric gliomas was recently studied by Clement et al. [65]. Immunohistochemical analyses identified IGF1R staining in 47 out of 53 tumors, and nuclear IGF1R labelling was observed in 10/47 cases. IGF1R staining was mostly non-nuclear in low-grade tumors, while nuclear expression was predominant in high-grade gliomas. Survival was significantly longer in patients with gliomas having non-nuclear IGF1R localization than in patients with nuclear IGF1R tumors. Hence, intracellular IGF1R localization may help in stratifying pediatric glioma patients.

To gain new insights into the clinical significance of nuclear IGF1R, Aleksic et al. [66] conducted ChIP-Seq analyses aimed at assessing global chromatin occupancy. The authors identified IGF1R binding on the *jun* and *fam21* promoters in fresh prostate cancer samples containing abundant nuclear IGF1R. A correlation between IGF1R and *jun* expression was observed in malignant prostate epithelium, suggesting the existence of a potentially novel mechanism of carcinogenesis. The growth factor amphiregulin participates in the nuclear accumulation of IGF1R in lung adenocarcinoma by allowing the binding of IGF1R to importin-β1 [67]. This process was associated with cell cycle arrest via p21 upregulation. In addition, nuclear IGF1R prevented the induction of apoptosis induced by gefitinib, an EGFR tyrosine kinase inhibitor. Hence, the data indicate that nuclear IGF1R is involved in resistance to EGFR-directed therapy in lung cancer.

Nuclear IGF1R was shown to phosphorylate proliferating cell nuclear antigen (PCNA), a key player in the DNA damage tolerance pathway [68]. The co-localization of IGF1R with PCNA was particularly evident in areas exhibiting dysplasia and invasion. However, this interaction was often lost in tumors with low response to chemotherapy. It appears that accumulation of DNA damage leads to abrogation of the physical interplay between IGF1R and PCNA as the tumor progresses, with ensuing reduction in genomic stability.

Given the potential role of IGF1R as a therapeutic target in oncology, it was of translational relevance to assess the impact of nuclear IGF1R on the clinical outcome. Using an immunohistochemical approach, Asmane et al. [69] demonstrated that exclusive nuclear IGF1R immunoreactivity (compared with cytoplasmic, or nuclear+cytoplasmic staining) was correlated with a better progression-free survival and overall survival in patients with unresectable or metastatic soft tissue sarcomas, Ewing sarcoma, and osteosarcoma treated with IGF1R monoclonal antibodies. In contrast, nuclear IGF1R correlated with poor survival in patients with metastatic colorectal cancer [64]. Chemo-resistant colon cancer cell lines had higher concentrations of nuclear IGF1R, and IGF1R translocation was enhanced by IGF1R blocking antibody ganitumab. The data are therefore in agreement with the view that nuclear IGF1R might have a role in the development of resistance to targeted or other therapies.

The prognostic value of breast-cancer-specific nuclear InsR was investigated in tissue microarrays from 900 patients with primary invasive breast cancer [70]. Nuclear InsR was assessed by immunohistochemistry and correlated with ER status, body mass index and clinical outcome. Patients were followed for up to 11 years. Data indicate that nuclear InsR was detected in 24% of patients and increased as a function of the time elapsed between surgery and staining. Nuclear InsR presence conferred higher risk of recurrence in ER+ tumors but lower risk in ER- cancers. Analysis of the data led to the conclusion that nuclear InsR may have prognostic value among normal weight patients with ER+ tumors and in obese or overweight patients with ER- tumors.

## 14. The Role of Nuclear InsR/IGF1R in Metabolism

Genome-wide analysis was recently employed to identify genes and signaling pathways affected by nuclear InsR and, more specifically, to evaluate the impact of this nuclear presence on insulin function [71]. ChIP-seq analysis revealed that InsR was predominantly localized to gene promoters, with robust enrichment for insulin-related activities. For example, genes involved in metabolism of lipids and lipoproteins, fatty acid and ketone body metabolism, and metabolism of amino acid derivatives, among other functions, were among the genes with the strongest InsR binding to promoter elements. In terms of association between InsR-bound promoters and disease categories, there was a strong correlation to diabetes and other disorders linked to insulin function, including Alzheimer’s disease and neurodegeneration.

Evidence that the nuclear InsR pathway is functionally involved in conditions associated with insulin resistance was provided by animal experiments showing a reduction in the levels of chromatin-bound InsR in an ob/ob mouse, a model of insulin resistance [71]. Of interest, functional analyses of InsR-bound promoters revealed a much larger representation of genes involved in lipid metabolism compared with genes involved in carbohydrate metabolism. These results are in agreement with the linkage between insulin resistance and dyslipidemia, a typical component of the metabolic syndrome.

In mechanistic terms, a strong physical interaction between InsR and transcriptional co-regulator HCF-1 was detected. HCF-1 knockout prevented InsR binding to DNA, suggesting that this transcription factor is directly responsible for InsR binding to chromatin. Taken together, nuclear InsR has a key role in the regulation of gene expression, and this molecular mechanism directly impinges upon the physiological and pathological actions of insulin.

Grave’s disease (GD) is an autoimmune disorder in which the thyroid gland is enlarged and becomes over-active. IGF1R was shown to be overexpressed by orbital fibroblasts derived from patients [72]. In addition, IGF1, as well as GD-derived IgG, led to IGF1R accumulation in the cell nucleus of GD fibroblasts, where it was found in association with chromatin. This process required ADAM17, a membrane-associated metalloproteinase. In contrast, nuclear IGF1R redistribution was not detected in control orbital fibroblasts. The significance of this specific IGF1R re-localization process in the context of GD orbital disease is at present unknown.

## 15. Conclusions

The identification of InsR and IGF1R in the nucleus of normal and cancer cells has generated a significant level of interest in recent years [73,74]. Curiosity was fueled by fundamental questions in the area of biological regulation as well as by more applied facets in clinical oncology. While nuclear insulin binding was first described more than 45 years ago, the recognition that classical cell-surface tyrosine kinase receptors can migrate to the cell nucleus and function as bona fide transcription factors stood against firmly established dogmas in signal transduction. Once this conceptual barrier fell, it became relevant to question the implications of these inverse (i.e., from nucleus to cell-surface) signaling paths of InsR/IGF1R.

The transcriptional role of InsR/IGF1R is not a trivial one. The ability of these important receptors to regulate gene expression at a genomic level provides a further layer of biological control. Moreover, and given the capacity of these receptors to sense (and respond to) ambient concentrations of insulin and IGF1, it is expected that the novel nuclear roles of InsR/IGF1R might be of major metabolic relevance.

Finally, the role of nuclear InsR/IGF1R in cancer biology is of exceptional translational relevance [75]. A better understanding of the complex mechanisms responsible for InsR/IGF1R transport to the nucleus and identification of InsR/IGF1R interactors might help to optimize target-directed therapies in oncology.

## Figures and Tables

**Table 1:** Nuclear IGF1R interactors in breast cells. Nuclear extracts of benign MCF10A cells were immunoprecipitated with an IGF1R antibody, after which mass spectroscopy analysis was conducted to identify candidate IGF1R interactors.

Gene Names	Protein Names	Student’s *t*-Test Difference	Student’s *t*-Test *p*-Value
ALDH18A1	Delta-1-pyrroline-5-carboxylate synthase	−6.61981074	0.000628706
TJP2	Tight junction protein ZO-2	−6.070006053	1.25 × 10^−8^
SIPA1L1	Signal-induced proliferation-associated 1-like protein 1	−5.783857346	9.09 × 10^−7^
RPS29	40S ribosomal protein S29	−5.609598796	3.73 × 10^−5^
VAPB	Vesicle-associated membrane protein-associated protein B/C	−4.180510203	6.64 ×10^−6^
TJP1	Tight junction protein ZO-1	−4.132112503	0.005198435
C17orf85	Uncharacterized protein C17orf85	−4.00877889	0.001092293
NUP88	Nuclear pore complex protein Nup88	−3.874612808	5.29 × 10^−6^
IGF1R	Insulin-like growth factor 1 receptor	−3.842482885	0.000196745
FAM162A	Protein FAM162A	−3.818694433	3.46 × 10^−6^
SPRR1B	Cornifin-B	−3.621960322	0.000569671
TJAP1	Tight junction-associated protein 1	−3.406965892	0.000618073
TUBB	Tubulin beta chain	−3.353492101	0.000981451
RAP1A	Ras-related protein Rap-1A	−3.285950343	7.10 × 10^−5^
NOM1	Nucleolar MIF4G domain-containing protein 1	−3.033599218	3.15 × 10^−5^
PABPC1	Polyadenylate-binding protein 1	−2.579547246	0.000578437
ZNF687	Zinc finger protein 687	−2.193822225	0.001073949
SART1	U4/U6.U5 tri-snRNP-associated protein 1	−0.605529785	0.001747125
NHP2L1	NHP2-like protein 1	−0.575263341	0.00718376

## Data Availability

No new data were created or analyzed in this study. Data sharing is not applicable to this article.
